# Effect of storage and DNA extraction method on 16S rRNA-profiled fecal microbiota in Japanese adults

**DOI:** 10.3164/jcbn.18-84

**Published:** 2018-12-13

**Authors:** Yuki Kawada, Yuji Naito, Akira Andoh, Motoyuki Ozeki, Ryo Inoue

**Affiliations:** 1 Laboratory of Animal Science, Department of Agriculture and Life Science, Kyoto Prefectural University, 1-5 Hangi-cho, Shimogamo, Sakyo-ku, Kyoto 606-8522, Japan; 2 Department of Molecular Gastroenterology and Hepatology, Kyoto Prefectural University of Medicine, Kajii-cho, Kamigyo-ku, Kyoto 602-8566, Japan; 3 Department of Medicine, Shiga University of Medical Science, Seta Tsukinowa-cho, Otsu, Shiga 520-2192, Japan; 4 Department of Informatics and Mediology, Mukogawa Women’s University, 6-46 Ikebiraki-cho, Nishinomiya, Hyogo 663-8558, Japan

**Keywords:** fecal microbiota, 16S rRNA-based profiling, fecal storage, bacterial DNA extraction

## Abstract

The effect of two factors, storage and the bacterial DNA extraction method, that potentially affect the 16S rRNA-based profiling of the microbiota in the feces of Japanese adults, were evaluated. Profiles of the microbiota in feces stored in DESS (DMSO-EDTA-salt solution) for 1, 2 and 3 weeks at room temperature, and for 3 weeks at 4°C were compared with those in fresh feces and feces stored in guanidine thiocyanate solution for 3 weeks at 4°C. None of the storage variables (preservation solution, temperature and duration) considerably affected α- and β-diversity of the fecal microbiota and OTU profiles. Regarding the bacterial DNA extraction methods, four were evaluated; A) silica membrane DNA purification combined with bead-beating bacterial disruption, B) magnetic bead DNA purification combined with bead-beating bacterial disruption, C) manual DNA purification using phenol-chloroform and ethanol precipitation combined with enzymatic bacterial lysis, and D) DNA extraction by a commercially available DNA stool kit. While methods A, B, and C did not markedly affect α- and β-diversity of the fecal microbiota and the OTU profiles, method D noticeably altered both α- and β-diversity. In addition, method D caused significant changes in the abundance of two predominant genera; *Bacteroides* and *Bifidobacterium*.

## Introduction

Human gastrointestinal tract harbors a dense microbial population known as the gut microbiota. The gut microbiota can significantly affect the metabolic and immunological functions of the host as demonstrated by previous experiments using germ-free animals.^(1)^ Moreover, accumulating evidence shows that the gut microbiota plays important roles not only in gastrointestinal disorders such as Crohn’s disease and ulcerative colitis^(2)^ but also in systemic chronic disorders such as obesity^(3)^ and cardiovascular disease.^(4)^

Deep sequencing of highly conserved regions in the 16S ribosomal RNA (rRNA) gene permits a comprehensive and comparative analysis of the commensal gut microbiota including non-cultivable bacteria.^(5)^ For instance, this analytical technique has provided evidence that shows that abundance of specific bacterial taxa in disease subjects differs from that in healthy individuals. As a result, deep sequencing is now widely popular, which has helped generate massive sequencing data of the 16S rRNA gene in the human gut microbiota including those obtained when assessing diseased versus healthy subjects^(6)^ and pre- and post-administration of several treatments.^(7)^ However, worldwide laboratories use different methods to generate sequencing data, which can pose a problem when conducting meta-analysis of multiple datasets created by different research groups. Thus, it is critical to elucidate the effect of different DNA analysis methodology can cause on samples and consequently, on the resulting data.

Feces are the most commonly used samples to evaluate the composition of gut microbiota because they can be easily collected in a non-invasive manner. Nonetheless, it is recognized that there are other factors affecting the profile analysis of the microbiota in fecal samples such as storage (including preservation solution, temperature and duration)^(8,9)^ and the bacterial DNA extraction method (e.g., mechanical or enzymatic lysis).^(10,11)^ Although several studies have evaluated the effects of these factors on the 16S rRNA-based profiles of the fecal microbiota, none was conducted in Japan and hence analyzed samples were not from Japanese subjects.^(8–12)^ In that context, Nishijima *et al.*^(13)^ reported that a unique characteristic of the gut microbiota of Japanese people represented by a high abundance of *Bifidobacterium*, which is not found in the microbiota of other ethnic groups evaluated. It is important to underline that the abundance analysis of *Bifidobacterium* can be highly affected by the DNA extraction method.^(10)^ With respect to sample storage, Nishimoto *et al.*^(14)^ and Hosomi *et al.*^(15)^ used a guanidine thiocyanate solution to store fecal samples from Japanese subjects. These workers reported that, at room temperature, the guanidine thiocyanate solution provided acceptable stability to the fecal microbiota. However, although robustness of the gut microbiota after a 2-month span^(14)^ and optimization of the bacterial DNA extraction method from samples stored in guanidine thiocyanate solution^(15)^ were assayed, the aforementioned work evaluated the effect of sample storage on the fecal microbiota only indirectly. Thus, it is deemed necessary to investigate the effect of storage conditions on samples in more detail.

In the present study, we evaluated two factors that could potentially affect the 16S rRNA-based profiling of fecal microbiota: 1) storage (including preservation solution, temperature and duration) and 2) the method of bacterial DNA extraction from feces.

For the storage assay, DESS (DMSO-EDTA-salt solution) was compared with the guanidine thiocyanate solution because they are equally regarded as acceptable media for fecal preservation^(16)^ As for the bacterial DNA extraction methods, four commonly used in Japan were evaluated; A) silica membrane-based DNA purification combined with bead-beating bacterial disruption, B) magnetic bead-based DNA purification combined with bead-beating bacterial disruption, C) manual DNA purification using phenol-chloroform and ethanol precipitation combined with enzymatic bacterial lysis, and D) commercially available DNA extraction by Qiagen.

## Materials and Methods

### Sample collection and storage

Feces were sampled at Kyoto Prefectural University from five healthy adult volunteers (3 males: 23 years old; 2 females: 22 years old). The experiment was approved by the Ethical Committee of Kyoto Prefectural University and conducted as per their guidelines (approval number: 132). Written informed consent was obtained from all participants.

Fecal samples were collected using a stool collection brush and storage tube set (Wako Pure Chemicals, Osaka, Japan). For evaluation of storage, feces were collected in sextuplicate from each subject. The preservation solution in each storage tube and storage duration are listed in Table 1. For evaluation of the bacterial DNA extraction method, fecal samples were collected from each subject in four DESS-containing tubes.

### DNA extraction

For evaluation of storage, bacterial DNA was extracted from samples using a QuickGene DNA tissue kit (KURABO, Osaka, Japan), as previously described.^(17)^

For evaluation of the DNA extraction method, DNA was extracted from samples using four different methods. As method A, the QuickGene DNA tissue kit S (silica membrane-based purification combined with bead-beating bacterial disruption) was used. As method B, a combined method of a Maxwell RSC blood DNA kit for purification (magnetic bead-based purification; Promega, Tokyo Japan) and bead-beating bacterial disruption was used. As method C, enzyme-based lysing and purification by phenol-chloroform and ethanol precipitation method^(18)^ were used. As method D, a combined method of a QIAamp DNA Stool Mini Kit (Qiagen, Tokyo, Japan) and bead-beating bacterial disruption^(19)^ was used.

### Deep sequencing of 16S rRNA genes and sequence data analysis

Library preparation including amplification by PCR of the V3–V4 region of the 16S rRNA gene was carried out exactly as previously described by Inoue *et al.*^(20)^ Deep sequencing was conducted using a MiSeq System (Illumina, Tokyo, Japan). Sequencing data analysis obtained was carried out exactly as previously described by Inoue *et al.*^(20)^

### Statistical analysis

α-Diversity indices Chao1 and Shannon were calculated by the R phyloseq package and statistically analyzed by the Kruskal-Wallis test followed by the Steel-Dwass posthoc test. β-Diversity was estimated based on Bray-Curtis distances and assayed by a principal coordinate analysis (PCoA) using phyloseq. The Bray-Curtis distance between the samples was statistically analyzed by a permutational multivariate analysis of variance (PERMANOVA). The relative abundance (%) of bacteria in the gut microbiota between groups was statistically compared by ANOVA, followed by the Tukey-Kramer posthoc test using the Benjamini-Hochberg false discovery rate correction (STAMP software).^(21)^ For α and β-diversity, differences were considered significant when *p*<0.05. For the relative abundance of bacteria, differences were considered significant when *p*<0.05 and* q*<0.1.

## Results

### Effect of storage

**Fig. 1:**
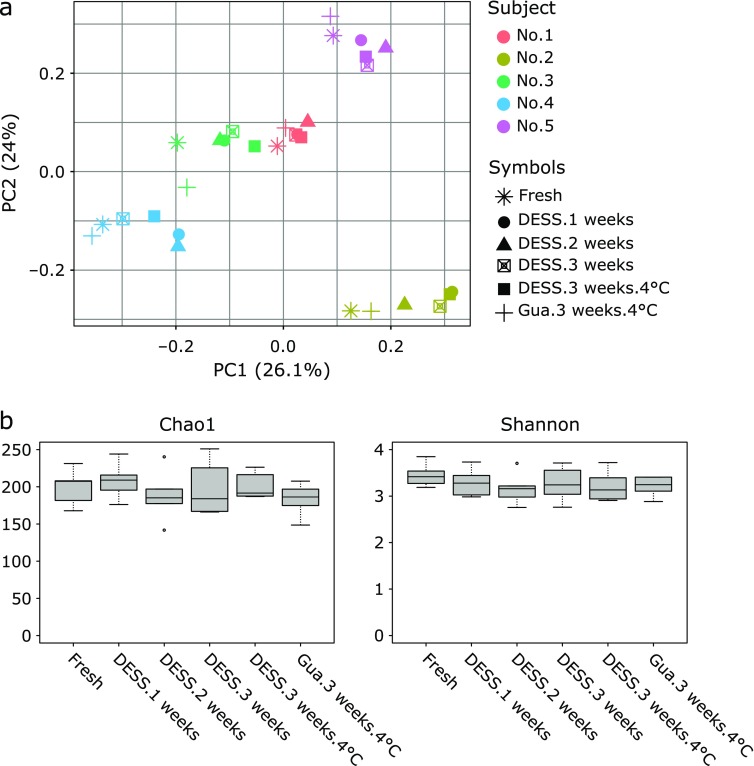
Comparative analysis of the fecal microbiota in fresh and stored samples. (a) Bray-Curtis PCoA. No significant differences were detected between fresh and stored samples (*p*>0.05, PERMANOVA). (b) Chao1 and Shannon indices of the fresh and stored samples. No significant differences were detected between fresh and stored samples (*p*>0.05, Kruskal-Wallis test).

**Fig. 2:**
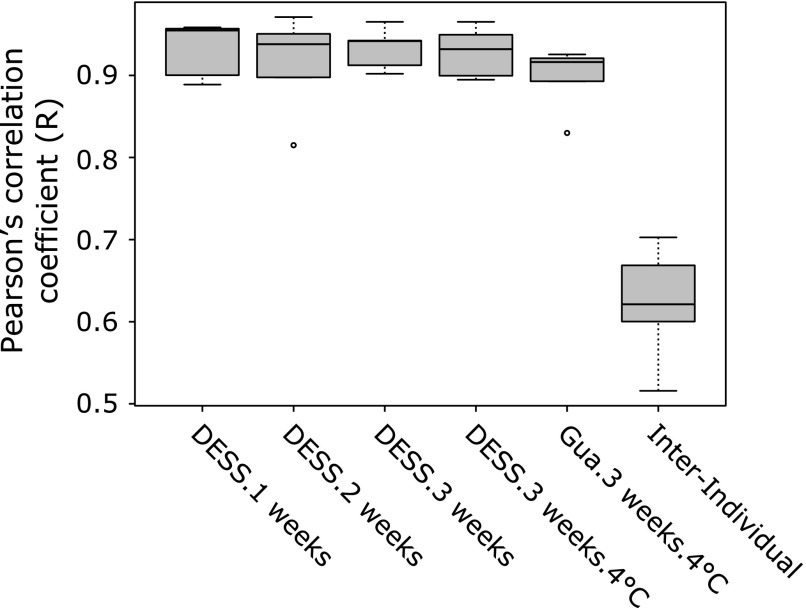
Correlation of OTU profiles in fresh and stored samples. Pearson’s correlation coefficient (R) between fresh and stored samples within individuals (Intra-Individual) was calculated. The correlation coefficient between fresh samples from a single individual (Inter-Individual) was also calculated to estimate difference in the fecal microbiota between individuals.

Regardless of the storage duration, temperature and solution, samples obtained from a single individual clustered together and only slight variations of the storage methods were observed within individuals (*p*>0.05; Fig. 1a). Moreover, independently of the storage method, the mean value of Pearson’s correlation coefficient of OTU profiles between fresh and stored samples within individuals (intra-individual) was about 0.90 or greater, but between individual fresh samples (inter-individual) it was 0.62 (Fig. 2).

**Fig. 3:**
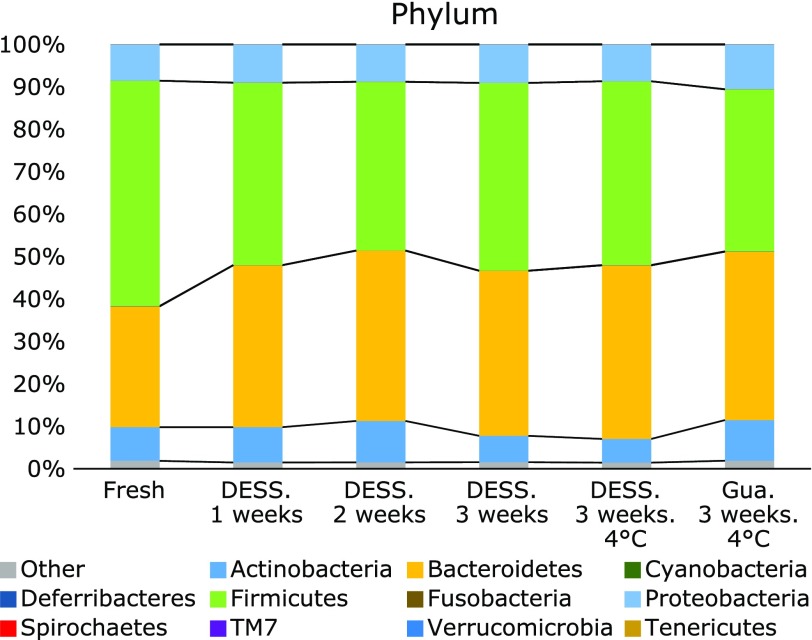
Taxonomic composition of the fecal microbiota in fresh and stored samples at phylum level.

At phylum level, regardless of the storage method, abundance of Bacteroidetes was approximately 10% higher in stored samples than in fresh samples, (*p*<0.05, *q*<0.1; Fig. 3). Conversely, abundance of Firmicutes was smaller in all stored samples than in fresh samples. The difference in abundance of Firmicutes between fresh samples and samples stored in DESS for 2 weeks at room temperature, and between fresh samples and samples stored in guanidine thiocyanate solution for 3 weeks at 4°C was significant (*p*<0.05, *q*<0.1; Fig. 3).

At genus level, a significant effect of storage was observed on two minor genera; *Ochrobactrum* and *Peptoniphilus* (Supplemental Table 1*). Indeed, except for the samples stored in DESS for 2 weeks at room temperature, abundance of *Ochrobactrum* was significantly higher in stored samples. Regarding the abundance of *Peptoniphilus*, it was unusually higher in samples stored in guanidine thiocyanate medium for 3 weeks at 4°C, when compared with other samples including fresh samples. α-Diversity in the samples was not affected by storage (Fig. 1b).

### Effect of bacterial DNA extraction methods

**Fig. 4:**
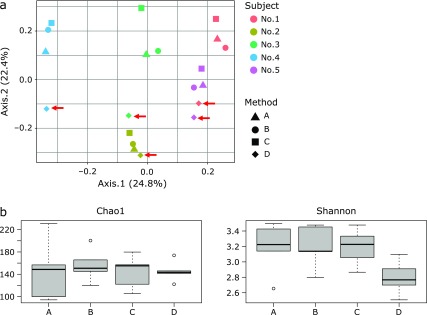
Comparative analysis of the fecal microbiota in samples from which DNA was extracted by four different methods. (a) Bray-Curtis PCoA. No significant differences were found between fresh and stored samples (*p*>0.05, PERMANOVA). The samples extracted by method D are indicated with arrows. (b) Chao1 and Shannon indices of fresh and stored samples. No significant differences were found between fresh and stored samples (*p*>0.05, Kruskal-Wallis test).

**Fig. 5:**
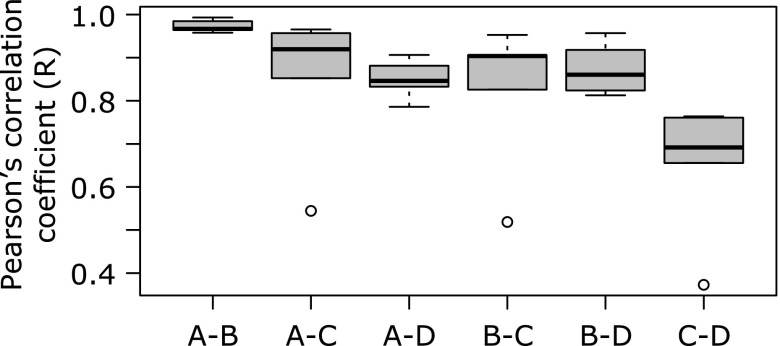
Correlation of OTU profiles between DNA extraction methods. Pearson’s correlation coefficient (R) between extraction methods was calculated.

 Regardless of the DNA extraction method, samples obtained from a single individual clustered together, but samples extracted by method D (Qiagen stool mini kit) were distantly located from samples extracted by the other three methods (PERMANOVA, *p*>0.05; Fig. 4a). Pearson’s correlation coefficient of OTU profiles was the highest between methods A and B (0.97 ± 0.01), and approximately 0.85 between the other methods except between method C and D (Fig. 5), which was markedly low (median 0.69).

**Fig. 6:**
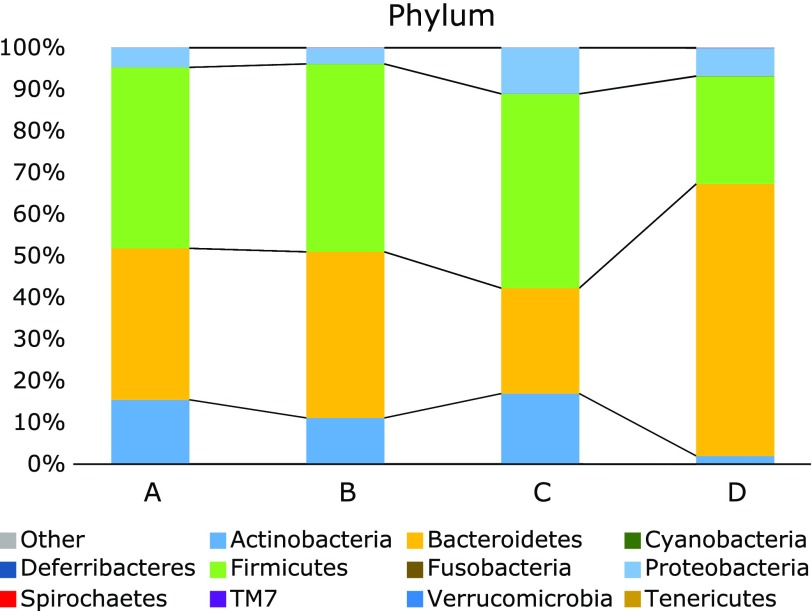
Taxonomic composition of the fecal microbiota at phylum level in samples from which DNA was extracted by four different methods.

At phylum level, abundance of Actinobacteria and Firmicutes was significantly lower in samples extracted by method D compared with samples extracted by other three methods (*p*<0.05, *q*<0.1; Fig. 6). In contrast, samples extracted by method D showed an abundance of Bacteroidetes significantly higher than that in samples extracted by the other three methods (*p*<0.05, *q*<0.1). Nonetheless, abundance of Bacteroidetes was significantly lower in samples extracted by method C than that in samples extracted by methods A and B (Fig. 6).

**Fig. 7:**
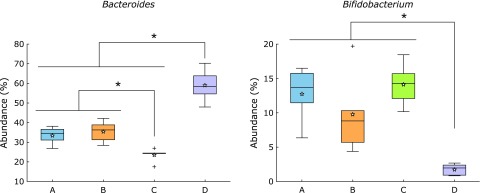
Abundance of bacterial genera that significantly differed between DNA extraction methods. *p<0.05, *q*<0.1 by ANOVA followed by the Tukey-Kramer posthoc test using the Benjamini-Hochberg false discovery rate correction. Stars in each box indicate the mean value.

At genus level, abundance of two predominant genera; *Bacteroides* and *Bifidobacterium* was affected by the extraction method, particularly by method D (Fig. 7, Supplemental Table 2*). Abundance of genus *Bacteroides* in samples extracted by method D was higher than that in samples extracted by the other three methods. By contrast, abundance of genus *Bifidobacterium* in samples extracted by method D was significantly lower than that in samples extracted by the other three methods. In addition, abundance of *Bacteroides* was the lowest in samples extracted by method C.

An effect of the extraction method was detected on α-diversity Shannon index but not on Chao1 index (Fig. 4b). Samples extracted by method D showed a non-significantly lower Shannon index than did samples extracted by the other three methods.

## Discussion

In gut microbiota research, deep sequencing of the 16S rRNA gene has become a common analytical method and thus, it is used in many laboratories around the world as well as in Japan. However, differences in the methods used by these laboratories to generate the sequencing data can make the meta-analysis of generated datasets difficult to achieve. In the present study, the effects of storage and the method of extraction of bacterial DNA from feces on the 16S rRNA-profiled microbiota were evaluated to establish if differences caused by these factors could alter microbiota profiles that in turn could significantly affect meta-analysis.

Storage variables such as preservation solution, temperature and duration did not affect the overall profiles of the fecal microbiota, as the PCoA analysis clearly showed that stored samples closely clustered with fresh samples (Fig. 1a) and that OTU profiles of the former showed a high correlation with those of the latter (>0.90) (Fig. 2). In addition, no effect of storage was found on α-diversity Chao1 and Shannon indices (Fig. 1b). Therefore, it can be postulated that in terms of the overall structure of the microbiota such as α-diversity or β-diversity, fecal samples stored even at room temperature in either DESS or guanidine thiocyanate solution for a maximum of 3 weeks, are likely to show robustness similar to that of fresh samples or those stored in different storage media. However, caution should be taken when comparing abundance of bacterial taxa in fresh and stored samples. Indeed, in the present study, abundance of Bacteroidetes at phylum level significantly decreased in stored samples when compared with that in fresh samples (Fig. 3), although abundance of Bacteroidetes in stored samples was unaffected by storage. At genus level, however, abundance of two minor genera, which was about 1% or less, was affected by storage (Supplemental Table 1*). Thus, these results seem to suggest that comparison of abundance of bacterial taxa can be safely made between different stored samples.

Differences in the bacterial DNA extraction methods noticeably altered the fecal microbiota profiles. For example, extraction methods A, B and C hardly affected the overall profiles, as per α-diversity and β-diversity of the fecal microbiota (Fig. 4). However, method D seemed to cause considerable differences in the overall profiles of the fecal microbiota. This effect was reflected by the way samples extracted by method D were distantly positioned in the PCoA plot (Fig. 4a) from samples extracted by the other three methods. In addition, Shannon index was noticeably lower in samples extracted by method D when compared with those extracted by other methods (Fig. 4b).

The effect of method D was observed on the abundance of the bacterial taxa, which was on predominant two genera, *Bacteroides* and *Bifidobacterium* (Fig. 7). Abundance of three phyla, Actinobacteria (to which genus *Bifidobacterium* belongs), Bacteroidetes (to which genus *Bacteroides* belongs) and Firmicutes was also significantly affected by method D (Fig. 6). Furthermore, abundance of phylum Bacteroidetes and genus *Bacteroides* in samples extracted by method C was significantly lower than that in the samples extracted by methods A and B. These results indicate that abundance of bacterial taxa in samples from which DNA was extracted by two different methods is comparable depending on the DNA method used. For example, samples extracted by method A and B can be compared, whereas those extracted by methods C and D cannot (Fig. 5). Interestingly, methods A and B used the same bacterial disruption step and hence it is not surprising that the results from these two extraction methods were highly correlated. Thus, it seems that unlike DNA purification, differences in bacterial lysis/disruption can cause effects on the fecal microbiota profiles, as it was clearly demonstrated by the elimination of the bead-beating processing in a previous study.^(10)^

In conclusion, the present study demonstrated that fecal storage for 3 weeks in DESS or guanidine thiocyanate solution either at room temperature or 4°C does not alter the microbiota profiles in fecal samples from Japanese subjects, as per the analysis of α-diversity, β-diversity and abundance of bacterial taxa. In addition, although the bacterial DNA extraction methods affect the profiles of the fecal microbiota, the results obtained from three of the four methods evaluated suggest that the effects may be acceptable for comparison of α-diversity and β-diversity. Nonetheless, the results obtained after using method D seem to suggest that assessment of the effect of the extraction method is critical prior to conducting meta-analyses of sequencing data. Phylum Bacteroidetes and genus *Bacteroides* seem to be more affected by both storage and the DNA extraction methods. These phylum and genus are predominant in the fecal microbiota of the Japanese population^(22)^ and it is reported that abundance of genus Bacteroides can be different in some disease subjects compared with healthy individuals.^(23–25)^ Therefore, meta-analysis studies involving abundance of bacterial taxa should be carefully designed, especially when data are obtained using different DNA extraction methods.

## Author Contributions

Naito Y, Andoh A and Inoue R conceived this experiment; Kawada Y and Inoue R collected feces; Kawada Y performed analysis of fecal bacteria; Kawada Y, Ozeki M and Inoue R analyzed data; Kawada Y and Inoue R were involved in editing the manuscript. All authors discussed the results and commented on the manuscript.

## Figures and Tables

**Table 1:** Sample strage conditions for the evaluation of storage method

Tube	Buffer contained	Storage duration	Storage temperature
1	Lysis buffer for QuickGene DNA tissu kit	Fresh^†^	4°C
2	Guanidine thiocyanate^‡^	3 weeks	4°C
3	DESS	3 weeks	4°C
4	DESS	1 week	Room temperature
5	DESS	2 weeks	Room temperature
6	DESS	3 weeks	Room temperature

^†^DNA was extracted within 12 h of sample collection, ^‡^Obtained from Techo Suruga Laboratory (Shizuoka, Japan).

## References

[1] Umesaki Y, Setoyama H (2000). Structure of the intestinal flora responsible for development of the gut immune system in a rodent model. Microbes Infect.

[2] Morgan XC, Tickle TL, Sokol H (2012). Dysfunction of the intestinal microbiome in inflammatory bowel disease and treatment. Genome Biol.

[3] Turnbaugh PJ, Ley RE, Mahowald MA, Magrini V, Mardis ER, Gordon JI (2006). An obesity-associated gut microbiome with increased capacity for energy harvest. Nature.

[4] Koeth RA, Wang Z, Levison BS (2013). Intestinal microbiota metabolism of L-carnitine, a nutrient in red meat, promotes atherosclerosis. Nat Med.

[5] Hiergeist A, Gläsner J, Reischl U, Gessner A (2015). Analyses of intestinal microbiota: culture versus sequencing. ILAR J.

[6] Inoue R, Sakaue Y, Sawai C (2016). A preliminary investigation on the relationship between gut microbiota and gene expressions in peripheral mononuclear cells of infants with autism spectrum disorders. Biosci Biotechnol Biochem.

[7] Otsuka T, Sugimoto M, Inoue R (2017). Influence of potassium-competitive acid blocker on the gut microbiome of *Helicobacter pylori*-negative healthy individuals. Gut.

[8] Carroll IM, Ringel-Kulka T, Siddle JP, Klaenhammer TR, Ringel Y (2012). Characterization of the fecal microbiota using high-throughput sequencing reveals a stable microbial community during storage. PLoS One.

[9] Fouhy F, Deane J, Rea MC (2015). The effects of freezing on faecal microbiota as determined using MiSeq sequencing and culture-based investigations. PLoS One.

[10] Santiago A, Panda S, Mengels G (2014). Processing faecal samples: a step forward for standards in microbial community analysis. BMC Microbiol.

[11] Yuan S, Cohen DB, Ravel J, Abdo Z, Fomey LJ (2012). Evaluation of methods for the extraction and purification of DNA from the human microbiome. PLoS One.

[12] Kuczynski J, Lauber CL, Walters WA (2011). Experimental and analytical tools for studying the human microbiome. Nat Rev Genet.

[13] Nishijima S, Suda W, Oshima K (2016). The gut microbiome of healthy Japanese and its microbial and functional uniqueness. DNA Res.

[14] Nishimoto Y, Mizutani S, Nakajima T (2016). High stability of faecal microbiome composition in guanidine thiocyanate solution at room temperature and robustness during colonoscopy. Gut.

[15] Hosomi K, Ohno H, Murakami H (2017). Method for preparing DNA from feces in guanidine thiocyanate solution affects 16S rRNA-based profiling of human microbiota diversity. Sci Rep.

[16] Gray MA, Pratte ZA, Kellogg CA (2013). Comparison of DNA preservation methods for environmental bacterial community samples. FEMS Microbiol Ecol.

[17] Matsumoto M, Inoue R, Tsuruta T, Hara H, Yajima T (2009). Long-term oral administration of cows’ milk improves insulin sensitivity in rats fed a high-sucrose diet. Br J Nutr.

[18] Kim SW, Suda W, Kim S (2013). Robustness of gut microbiota of healthy adults in response to probiotic intervention revealed by high-throughput pyrosequencing. DNA Res.

[19] Mirsepasi H, Persson S, Struve C, Andersen LO, Petersen AM, Krogfelt KA (2014). Microbial diversity in fecal samples depends on DNA extraction method: easyMag DNA extraction compared to QIAamp DNA stool mini kit extraction. BMC Res Notes.

[20] Inoue R, Ohue-Kitano R, Tsukahara T (2017). Prediction of functional profiles of gut microbiota from 16S rRNA metagenomic data provides a more robust evaluation of gut dysbiosis occurring in Japanese type 2 diabetic patients. J Clin Biochem Nutr.

[21] Parks DH, Tyson GW, Hugenholtz P, Beiko RG (2014). STAMP: statistical analysis of taxonomic and functional profiles. Bioinformatics.

[22] Takagi T Naito Y Inoue R Differences in gut microbiota associated with age, sex, and stool consistency in healthy Japanese subjects. J Gastroenterol. 2018. DOI: 10.1007/s00535-018-1488-5 10.1007/s00535-018-1488-5 29926167

[23] Andoh A, Imaeda H, Aomatsu T (2011). Comparison of the fecal microbiota profiles between ulcerative colitis and Crohn’s disease using terminal restriction fragment length polymorphism analysis. J Gastroenterol.

[24] Andoh A, Nishida A, Takahashi K (2016). Comparison of the gut microbial community between obese and lean peoples using 16S gene sequencing in a Japanese population. J Clin Biochem Nutr.

[25] Ojima M, Motooka D, Shimizu K (2016). Metagenomic analysis reveals dynamic changes of whole gut microbiota in the acute phase of intensive care unit patients. Dig Dis Sci.

