# Supplementary material for: Effect of storage and DNA extraction method on 16S rRNA-profiled fecal microbiota in Japanese adults
Source: J Clin Biochem Nutr. 2018 Dec 13;64(2):106–11. doi: 10.3164/jcbn.18-84 (PMC6436037; doi:10.3164/jcbn.18-84)
Supplement: Supplemental Table 1 [file jcbn18-84st01.pdf]

Supplemental Table 1. Fecal microbiota composition at genus level in the fresh and stored samples

Phylum	Class	Order	Family	Genus	Fresh	DESS.1wk	DESS.2wk	DESS.3wk	DESS.3wk.F	Gua.3wk.F	*p* values	*q* values
Actinobacteria	Actinobacteria	Bifidobacteriales	*Bifidobacteriaceae*	*Bifidobacterium*	7.11 ± 2.30	7.35 ± 2.35	8.69 ± 2.61	5.10 ± 1.82	4.81 ± 1.67	8.76 ± 5.25	0.28	2.53
Actinobacteria	Actinobacteria	Actinomycetales	*Micrococcaceae*	*Rothia*	0.00 ± 0.00	0.00 ± 0.00	0.00 ± 0.00	0.00 ± 0.00	0.01 ± 0.02	0.00 ± 0.00	0.35	2.52
Actinobacteria	Actinobacteria	Bifidobacteriales	*Bifidobacteriaceae*	*Scardovia*	0.00 ± 0.00	0.00 ± 0.00	0.00 ± 0.00	0.00 ± 0.00	0.00 ± 0.00	0.00 ± 0.00	0.44	1.93
Actinobacteria	Actinobacteria	Actinomycetales	*Corynebacteriaceae*	*Corynebacterium*	0.00 ± 0.00	0.00 ± 0.00	0.00 ± 0.00	0.00 ± 0.00	0.00 ± 0.00	0.00 ± 0.00	0.46	1.95
Actinobacteria	Actinobacteria	Actinomycetales	*Propionibacteriaceae*	*Propionibacterium*	0.05 ± 0.04	0.03 ± 0.01	0.03 ± 0.01	0.03 ± 0.03	0.02 ± 0.02	0.04 ± 0.03	0.75	1.43
Actinobacteria	Actinobacteria	Actinomycetales	*Actinomycetaceae*	*Actinomyces*	0.02 ± 0.01	0.02 ± 0.02	0.03 ± 0.01	0.02 ± 0.02	0.01 ± 0.02	0.02 ± 0.01	0.85	1.45
Actinobacteria	Coriobacteriia	Coriobacteriales	*Coriobacteriaceae*	Unclassified	0.01 ± 0.01	0.03 ± 0.05	0.02 ± 0.04	0.02 ± 0.04	0.02 ± 0.04	0.00 ± 0.01	0.94	1.31
Actinobacteria	Coriobacteriia	Coriobacteriales	*Coriobacteriaceae*	*Eggerthella*	0.11 ± 0.10	0.09 ± 0.06	0.08 ± 0.09	0.09 ± 0.11	0.10 ± 0.08	0.06 ± 0.03	0.97	1.22
Actinobacteria	Coriobacteriia	Coriobacteriales	*Coriobacteriaceae*	*Collinsella*	0.62 ± 0.69	0.80 ± 0.73	0.87 ± 0.92	0.93 ± 1.17	0.59 ± 0.56	0.68 ± 0.94	0.99	1.11
Bacteroidetes	Bacteroidia	Bacteroidales	*Bacteroidaceae*	*Bacteroides*	24.17 ± 4.60	33.28 ± 3.38	36.38 ± 2.84	34.06 ± 5.01	35.77 ± 5.38	33.65 ± 4.34	0.01	0.24
Bacteroidetes	Bacteroidia	Bacteroidales	S24-7	Unclassified	0.47 ± 0.27	0.54 ± 0.26	0.19 ± 0.11	0.36 ± 0.30	0.26 ± 0.07	0.33 ± 0.09	0.19	2.44
Bacteroidetes	Bacteroidia	Bacteroidales	[*Paraprevotellaceae*]	[*Prevotella*]	0.08 ± 0.05	0.09 ± 0.04	0.04 ± 0.03	0.05 ± 0.05	0.05 ± 0.03	0.04 ± 0.02	0.35	2.35
Bacteroidetes	Bacteroidia	Bacteroidales	Unclassified	Unclassified	0.01 ± 0.01	0.00 ± 0.00	0.01 ± 0.01	0.00 ± 0.00	0.01 ± 0.00	0.01 ± 0.01	0.73	1.53
Bacteroidetes	Bacteroidia	Bacteroidales	*Porphyromonadaceae*	*Porphyromonas*	0.00 ± 0.00	0.00 ± 0.00	0.00 ± 0.00	0.00 ± 0.00	0.00 ± 0.00	0.00 ± 0.01	0.82	1.42
Bacteroidetes	Bacteroidia	Bacteroidales	[*Barnesiellaceae*]	Unclassified	0.19 ± 0.38	0.12 ± 0.23	0.12 ± 0.24	0.17 ± 0.33	0.20 ± 0.41	0.38 ± 0.75	0.96	1.26
Bacteroidetes	Bacteroidia	Bacteroidales	*Porphyromonadaceae*	*Parabacteroides*	1.17 ± 1.16	1.90 ± 1.92	1.72 ± 1.67	1.92 ± 1.92	1.71 ± 1.77	2.53 ± 3.13	0.96	1.25
Bacteroidetes	Bacteroidia	Bacteroidales	*Rikenellaceae*	Unclassified	0.28 ± 0.27	0.24 ± 0.22	0.27 ± 0.31	0.39 ± 0.50	0.44 ± 0.61	0.39 ± 0.40	0.97	1.22
Bacteroidetes	Bacteroidia	Bacteroidales	[*Odoribacteraceae*]	*Odoribacter*	0.16 ± 0.20	0.14 ± 0.19	0.18 ± 0.23	0.23 ± 0.29	0.21 ± 0.30	0.24 ± 0.38	0.99	1.07
Bacteroidetes	Bacteroidia	Bacteroidales	*Prevotellaceae*	*Prevotella*	1.91 ± 3.69	1.79 ± 3.49	1.19 ± 2.34	1.67 ± 3.27	2.25 ± 4.47	2.08 ± 4.10	1.00	1.01
Cyanobacteria	Chloroplast	Streptophyta	Unclassified	Unclassified	0.00 ± 0.00	0.00 ± 0.00	0.00 ± 0.00	0.01 ± 0.01	0.00 ± 0.00	0.00 ± 0.01	0.41	2.07
Deferribacteres	Deferribacteres	Deferribacterales	*Deferribacteraceae*	*Mucispirillum*	0.07 ± 0.06	0.05 ± 0.04	0.03 ± 0.02	0.05 ± 0.03	0.04 ± 0.02	0.07 ± 0.03	0.62	1.68
**Firmicutes**	**Clostridia**	**Clostridiales**	**[*Tissierellaceae*]**	***Peptoniphilus***	**0.00 ± 0.00**	**0.00 ± 0.00**	**0.00 ± 0.00**	**0.00 ± 0.00**	**0.00 ± 0.01**	**0.01 ± 0.01**	**<0.01**	**0.07**
Firmicutes	Clostridia	Clostridiales	*Lachnospiraceae*	Unclassified	6.91 ± 1.91	4.82 ± 1.28	3.71 ± 1.05	4.83 ± 0.89	5.03 ± 1.50	3.27 ± 0.75	0.01	0.26
Firmicutes	Bacilli	Bacillales	*Planococcaceae*	*Lysinibacillus*	0.00 ± 0.00	0.00 ± 0.00	0.00 ± 0.00	0.00 ± 0.00	0.00 ± 0.00	0.00 ± 0.00	0.09	1.84
Firmicutes	Bacilli	Lactobacillales	*Carnobacteriaceae*	*Granulicatella*	0.01 ± 0.01	0.00 ± 0.00	0.00 ± 0.00	0.00 ± 0.01	0.00 ± 0.00	0.00 ± 0.00	0.11	1.83
Firmicutes	Erysipelotrichi	Erysipelotrichales	*Erysipelotrichaceae*	*Coprobacillus*	0.07 ± 0.04	0.04 ± 0.03	0.03 ± 0.02	0.03 ± 0.02	0.05 ± 0.03	0.02 ± 0.02	0.22	2.42
Firmicutes	Bacilli	Lactobacillales	*Lactobacillaceae*	*Lactobacillus*	0.13 ± 0.05	0.14 ± 0.07	0.07 ± 0.04	0.07 ± 0.06	0.17 ± 0.14	0.16 ± 0.07	0.32	2.49
Firmicutes	Bacilli	Bacillales	[*Exiguobacteraceae*]	Unclassified	0.01 ± 0.01	0.00 ± 0.00	0.01 ± 0.01	0.00 ± 0.00	0.01 ± 0.00	0.00 ± 0.00	0.39	2.34
Firmicutes	Bacilli	Lactobacillales	*Streptococcaceae*	*Streptococcus*	1.29 ± 0.74	0.83 ± 0.45	0.69 ± 0.43	0.82 ± 0.51	0.68 ± 0.42	0.57 ± 0.29	0.40	2.25
Firmicutes	Clostridia	Clostridiales	*Lachnospiraceae*	*Blautia*	6.46 ± 3.34	3.97 ± 2.03	3.27 ± 1.58	3.73 ± 2.10	3.73 ± 2.18	3.75 ± 1.93	0.42	2.00
Firmicutes	Clostridia	Clostridiales	[*Tissierellaceae*]	*Anaerococcus*	0.00 ± 0.01	0.00 ± 0.00	0.00 ± 0.00	0.00 ± 0.00	0.00 ± 0.00	0.01 ± 0.02	0.42	1.95
Firmicutes	Erysipelotrichi	Erysipelotrichales	*Erysipelotrichaceae*	Unclassified	1.00 ± 0.67	0.57 ± 0.37	0.56 ± 0.42	0.58 ± 0.53	0.50 ± 0.32	0.36 ± 0.24	0.49	1.97
Firmicutes	Clostridia	Clostridiales	*Lachnospiraceae*	*Oribacterium*	0.00 ± 0.00	0.00 ± 0.00	0.00 ± 0.00	0.00 ± 0.00	0.00 ± 0.00	0.00 ± 0.01	0.49	1.90
Firmicutes	Clostridia	Clostridiales	*Lachnospiraceae*	*Roseburia*	3.26 ± 1.78	1.81 ± 0.65	1.75 ± 0.77	2.04 ± 1.01	2.38 ± 1.34	2.03 ± 1.53	0.56	1.71
Firmicutes	Clostridia	Clostridiales	*Peptostreptococcaceae*	*Peptostreptococcus*	0.01 ± 0.01	0.00 ± 0.01	0.02 ± 0.03	0.00 ± 0.01	0.00 ± 0.00	0.00 ± 0.00	0.59	1.76
Firmicutes	Clostridia	Clostridiales	[*Tissierellaceae*]	*Parvimonas*	0.01 ± 0.01	0.01 ± 0.01	0.01 ± 0.01	0.00 ± 0.00	0.00 ± 0.00	0.01 ± 0.01	0.61	1.76
Firmicutes	Clostridia	Clostridiales	*Peptococcaceae*	Unclassified	0.00 ± 0.00	0.00 ± 0.00	0.00 ± 0.00	0.00 ± 0.00	0.00 ± 0.00	0.00 ± 0.00	0.62	1.73
Firmicutes	Bacilli	Lactobacillales	*Leuconostocaceae*	*Leuconostoc*	0.00 ± 0.00	0.00 ± 0.00	0.00 ± 0.00	0.00 ± 0.00	0.00 ± 0.00	0.00 ± 0.00	0.64	1.65
Firmicutes	Clostridia	Clostridiales	*Lachnospiraceae*	*Coprococcus*	4.05 ± 2.76	2.50 ± 1.82	2.03 ± 1.44	2.52 ± 1.78	1.98 ± 1.41	2.23 ± 2.10	0.68	1.63
Firmicutes	Clostridia	Clostridiales	*Ruminococcaceae*	*Clostridium*	0.01 ± 0.01	0.01 ± 0.01	0.01 ± 0.02	0.00 ± 0.01	0.01 ± 0.01	0.01 ± 0.01	0.69	1.63
Firmicutes	Bacilli	Lactobacillales	*Leuconostocaceae*	*Weissella*	0.00 ± 0.00	0.00 ± 0.01	0.00 ± 0.00	0.00 ± 0.00	0.00 ± 0.00	0.00 ± 0.00	0.72	1.61
Firmicutes	Clostridia	Clostridiales	[*Tissierellaceae*]	WAL_1855D	0.01 ± 0.01	0.00 ± 0.00	0.00 ± 0.00	0.00 ± 0.00	0.00 ± 0.00	0.01 ± 0.00	0.72	1.56
Firmicutes	Clostridia	Clostridiales	*Christensenellaceae*	Unclassified	0.01 ± 0.01	0.00 ± 0.00	0.00 ± 0.00	0.00 ± 0.01	0.01 ± 0.01	0.01 ± 0.01	0.73	1.50
Firmicutes	Bacilli	Bacillales	*Staphylococcaceae*	*Staphylococcus*	0.01 ± 0.01	0.01 ± 0.00	0.00 ± 0.00	0.01 ± 0.01	0.00 ± 0.01	0.01 ± 0.01	0.74	1.46
Firmicutes	Clostridia	Clostridiales	*Ruminococcaceae*	*Anaerotruncus*	0.02 ± 0.02	0.02 ± 0.01	0.01 ± 0.01	0.03 ± 0.03	0.01 ± 0.01	0.02 ± 0.02	0.74	1.44
Firmicutes	Clostridia	Clostridiales	*Eubacteriaceae*	*Pseudoramibacter_Eubacterium*	0.01 ± 0.01	0.00 ± 0.00	0.00 ± 0.00	0.00 ± 0.01	0.00 ± 0.01	0.01 ± 0.01	0.85	1.43
Firmicutes	Clostridia	Clostridiales	*Lachnospiraceae*	*Dorea*	0.94 ± 0.56	0.78 ± 0.60	0.83 ± 0.65	0.67 ± 0.56	0.74 ± 0.52	0.44 ± 0.38	0.85	1.42
Firmicutes	Clostridia	Clostridiales	*Lachnospiraceae*	[*Ruminococcus*]	3.80 ± 2.76	2.80 ± 1.91	2.38 ± 1.79	2.86 ± 2.16	2.40 ± 1.77	2.08 ± 1.22	0.87	1.41
Firmicutes	Clostridia	Clostridiales	*Ruminococcaceae*	*Butyricicoccus*	0.77 ± 0.44	0.65 ± 0.40	0.78 ± 0.53	0.55 ± 0.31	0.59 ± 0.30	0.50 ± 0.27	0.88	1.39
Firmicutes	Clostridia	Clostridiales	*Clostridiaceae*	SMB53	0.12 ± 0.07	0.17 ± 0.14	0.15 ± 0.15	0.17 ± 0.12	0.09 ± 0.07	0.18 ± 0.16	0.89	1.36
Firmicutes	Bacilli	Lactobacillales	*Streptococcaceae*	*Lactococcus*	0.03 ± 0.06	0.01 ± 0.01	0.02 ± 0.04	0.01 ± 0.01	0.02 ± 0.03	0.01 ± 0.02	0.91	1.36
Firmicutes	Clostridia	Clostridiales	*Lachnospiraceae*	*Clostridium*	0.40 ± 0.62	0.21 ± 0.30	0.18 ± 0.25	0.24 ± 0.32	0.31 ± 0.42	0.12 ± 0.14	0.92	1.34
Firmicutes	Bacilli	Bacillales	*Bacillaceae*	*Bacillus*	0.03 ± 0.06	0.02 ± 0.03	0.01 ± 0.02	0.02 ± 0.03	0.01 ± 0.02	0.03 ± 0.07	0.93	1.32
Firmicutes	Clostridia	Clostridiales	*Clostridiaceae*	*Clostridium*	1.03 ± 1.19	0.71 ± 0.73	0.59 ± 0.67	0.59 ± 0.64	0.50 ± 0.45	0.63 ± 0.76	0.94	1.29
Firmicutes	Clostridia	Clostridiales	*Ruminococcaceae*	*Faecalibacterium*	8.84 ± 3.31	10.09 ± 4.39	10.26 ± 4.89	11.02 ± 4.88	10.60 ± 4.19	8.54 ± 3.10	0.95	1.28
Firmicutes	Clostridia	Clostridiales	*Lachnospiraceae*	*Lachnospira*	0.99 ± 1.12	1.32 ± 1.50	1.24 ± 1.53	1.88 ± 2.19	1.94 ± 2.19	1.85 ± 2.37	0.97	1.23
Firmicutes	Clostridia	Clostridiales	*Veillonellaceae*	*Phascolarctobacterium*	0.29 ± 0.54	0.39 ± 0.77	0.47 ± 0.93	0.65 ± 1.29	0.61 ± 1.20	0.19 ± 0.37	0.98	1.20
Firmicutes	Clostridia	Clostridiales	*Ruminococcaceae*	Unclassified	1.73 ± 1.75	1.22 ± 1.08	1.34 ± 1.25	1.45 ± 1.34	1.31 ± 1.39	0.96 ± 0.84	0.98	1.19
Firmicutes	Erysipelotrichi	Erysipelotrichales	*Erysipelotrichaceae*	*Holdemania*	0.03 ± 0.03	0.02 ± 0.03	0.03 ± 0.04	0.03 ± 0.04	0.02 ± 0.03	0.02 ± 0.02	0.98	1.18
Firmicutes	Bacilli	Lactobacillales	*Enterococcaceae*	*Enterococcus*	0.05 ± 0.08	0.02 ± 0.04	0.04 ± 0.07	0.03 ± 0.04	0.04 ± 0.07	0.02 ± 0.03	0.98	1.16
Firmicutes	Bacilli	Gemellales	*Gemellaceae*	Unclassified	0.01 ± 0.01	0.01 ± 0.00	0.01 ± 0.01	0.01 ± 0.00	0.01 ± 0.01	0.01 ± 0.01	0.98	1.15
Firmicutes	Bacilli	Turicibacterales	*Turicibacteraceae*	*Turicibacter*	0.15 ± 0.17	0.25 ± 0.39	0.22 ± 0.29	0.20 ± 0.27	0.14 ± 0.21	0.27 ± 0.39	0.98	1.14
Firmicutes	Clostridia	Clostridiales	*Lachnospiraceae*	*Anaerostipes*	0.19 ± 0.20	0.14 ± 0.18	0.11 ± 0.12	0.16 ± 0.20	0.12 ± 0.14	0.19 ± 0.27	0.98	1.13
Firmicutes	Clostridia	Clostridiales	*Ruminococcaceae*	*Oscillospira*	1.24 ± 1.03	1.53 ± 1.96	1.60 ± 1.81	1.14 ± 1.09	1.51 ± 1.65	2.00 ± 2.09	0.98	1.12
Firmicutes	Clostridia	Clostridiales	[*Mogibacteriaceae*]	Unclassified	0.06 ± 0.08	0.04 ± 0.05	0.04 ± 0.07	0.04 ± 0.07	0.03 ± 0.05	0.04 ± 0.06	0.99	1.09
Firmicutes	Clostridia	Clostridiales	*Veillonellaceae*	*Megasphaera*	1.07 ± 2.04	0.83 ± 1.58	0.89 ± 1.75	0.91 ± 1.81	0.72 ± 1.40	0.38 ± 0.73	0.99	1.08
Firmicutes	Clostridia	Clostridiales	Unclassified	Unclassified	0.38 ± 0.24	0.32 ± 0.22	0.31 ± 0.26	0.39 ± 0.40	0.39 ± 0.24	0.37 ± 0.10	1.00	1.06
Firmicutes	Clostridia	Clostridiales	*Veillonellaceae*	*Dialister*	0.87 ± 1.03	0.63 ± 0.81	0.57 ± 0.77	0.67 ± 0.94	0.88 ± 1.26	0.81 ± 1.03	1.00	1.05
Firmicutes	Bacilli	Lactobacillales	*Lactobacillaceae*	*Pediococcus*	0.18 ± 0.31	0.13 ± 0.22	0.11 ± 0.20	0.11 ± 0.19	0.13 ± 0.25	0.13 ± 0.17	1.00	1.04
Firmicutes	Erysipelotrichi	Erysipelotrichales	*Erysipelotrichaceae*	[*Eubacterium*]	1.06 ± 1.86	0.72 ± 1.26	0.61 ± 1.12	0.67 ± 1.20	0.66 ± 1.17	0.76 ± 1.44	1.00	1.03
Firmicutes	Clostridia	Clostridiales	*Ruminococcaceae*	*Ruminococcus*	1.74 ± 2.28	1.68 ± 2.11	1.59 ± 2.03	1.20 ± 1.45	1.35 ± 1.53	1.75 ± 2.58	1.00	1.02
Firmicutes	Clostridia	Clostridiales	*Veillonellaceae*	*Veillonella*	3.76 ± 2.98	3.51 ± 2.47	3.16 ± 2.27	3.84 ± 2.78	3.63 ± 2.67	3.38 ± 3.09	1.00	1.00
Fusobacteria	Fusobacteriia	Fusobacteriales	*Fusobacteriaceae*	*Fusobacterium*	0.02 ± 0.03	0.04 ± 0.06	0.02 ± 0.04	0.02 ± 0.04	0.01 ± 0.02	0.04 ± 0.06	0.96	1.27
**Proteobacteria**	**Alphaproteobacteria**	**Rhizobiales**	***Brucellaceae***	***Ochrobactrum***	**0.04 ± 0.03**	**0.72 ± 0.33**	**0.03 ± 0.02**	**0.72 ± 0.33**	**0.92 ± 0.37**	**1.03 ± 0.37**	**<0.01**	**<0.01**
Proteobacteria	Alphaproteobacteria	Sphingomonadales	*Sphingomonadaceae*	*Sphingomonas*	0.00 ± 0.00	0.00 ± 0.00	0.00 ± 0.00	0.00 ± 0.00	0.00 ± 0.00	0.00 ± 0.00	0.36	2.28
Proteobacteria	Proteobacteria	Proteobacteria	*Proteobacteria*	*Proteobacteria*	0.83 ± 0.19	0.59 ± 0.11	0.59 ± 0.17	0.64 ± 0.22	0.62 ± 0.11	0.66 ± 0.27	0.49	1.83
Proteobacteria	Alphaproteobacteria	Unclassified	Unclassified	Unclassified	4.94 ± 1.49	3.88 ± 0.78	4.07 ± 1.35	4.03 ± 1.55	3.68 ± 0.67	5.17 ± 1.50	0.49	1.77
Proteobacteria	Alphaproteobacteria	Rickettsiales	*Mitochondria*	*Saprolegnia*	0.00 ± 0.00	0.00 ± 0.00	0.00 ± 0.00	0.00 ± 0.00	0.00 ± 0.00	0.00 ± 0.00	0.52	1.81
Proteobacteria	Gammaproteobacteria	Pseudomonadales	*Pseudomonadaceae*	*Pseudomonas*	0.00 ± 0.00	0.00 ± 0.00	0.00 ± 0.00	0.00 ± 0.00	0.00 ± 0.00	0.00 ± 0.00	0.53	1.79
Proteobacteria	Alphaproteobacteria	Rhizobiales	*Rhizobiaceae*	*Rhizobium*	0.00 ± 0.00	0.00 ± 0.00	0.00 ± 0.00	0.00 ± 0.00	0.00 ± 0.00	0.00 ± 0.01	0.55	1.78
Proteobacteria	Alphaproteobacteria	Rickettsiales	Unclassified	Unclassified	0.00 ± 0.00	0.00 ± 0.00	0.00 ± 0.01	0.00 ± 0.00	0.00 ± 0.00	0.00 ± 0.00	0.55	1.74
Proteobacteria	Deltaproteobacteria	Desulfovibrionales	*Desulfovibrionaceae*	Unclassified	0.00 ± 0.01	0.00 ± 0.00	0.00 ± 0.00	0.00 ± 0.00	0.00 ± 0.00	0.01 ± 0.01	0.62	1.64
Proteobacteria	Alphaproteobacteria	Rickettsiales	*Mitochondria*	Unclassified	0.51 ± 0.09	0.44 ± 0.10	0.45 ± 0.10	0.45 ± 0.09	0.47 ± 0.08	0.55 ± 0.17	0.66	1.63
Proteobacteria	Gammaproteobacteria	Enterobacteriales	*Enterobacteriaceae*	*Klebsiella*	0.00 ± 0.00	0.01 ± 0.02	0.00 ± 0.00	0.01 ± 0.01	0.00 ± 0.01	0.01 ± 0.02	0.71	1.63
Proteobacteria	Alphaproteobacteria	Rhizobiales	*Rhizobiaceae*	*Agrobacterium*	0.00 ± 0.01	0.00 ± 0.00	0.00 ± 0.00	0.00 ± 0.00	0.00 ± 0.00	0.00 ± 0.00	0.72	1.59
Proteobacteria	Gammaproteobacteria	Pasteurellales	*Pasteurellaceae*	Unclassified	0.00 ± 0.00	0.00 ± 0.01	0.00 ± 0.01	0.00 ± 0.00	0.00 ± 0.00	0.00 ± 0.00	0.73	1.48
Proteobacteria	Deltaproteobacteria	Desulfovibrionales	*Desulfovibrionaceae*	*Desulfovibrio*	0.02 ± 0.02	0.01 ± 0.01	0.02 ± 0.01	0.02 ± 0.02	0.01 ± 0.01	0.02 ± 0.01	0.80	1.46
Proteobacteria	Betaproteobacteria	Burkholderiales	*Alcaligenaceae*	*Sutterella*	1.05 ± 0.56	1.56 ± 0.77	1.78 ± 0.98	1.76 ± 0.85	1.41 ± 0.69	1.33 ± 1.00	0.81	1.46
Proteobacteria	Gammaproteobacteria	Pseudomonadales	*Moraxellaceae*	*Acinetobacter*	0.12 ± 0.10	0.09 ± 0.05	0.07 ± 0.05	0.10 ± 0.08	0.08 ± 0.05	0.13 ± 0.07	0.81	1.44
Proteobacteria	Deltaproteobacteria	Desulfovibrionales	*Desulfovibrionaceae*	*Bilophila*	0.11 ± 0.12	0.16 ± 0.19	0.19 ± 0.28	0.19 ± 0.26	0.30 ± 0.44	0.08 ± 0.07	0.87	1.40
Proteobacteria	Alphaproteobacteria	RF32	RF32	RF32	0.02 ± 0.03	0.01 ± 0.02	0.02 ± 0.05	0.02 ± 0.03	0.01 ± 0.02	0.05 ± 0.09	0.89	1.38
Proteobacteria	Betaproteobacteria	Burkholderiales	*Comamonadaceae*	*Delftia*	0.00 ± 0.00	0.00 ± 0.00	0.00 ± 0.00	0.00 ± 0.00	0.00 ± 0.00	0.00 ± 0.00	0.89	1.35
Proteobacteria	Gammaproteobacteria	Enterobacteriales	*Enterobacteriaceae*	*Escherichia*	0.67 ± 0.72	1.05 ± 0.91	0.96 ± 0.71	0.65 ± 0.54	0.66 ± 0.40	1.07 ± 1.18	0.92	1.33
Proteobacteria	Gammaproteobacteria	Pasteurellales	*Pasteurellaceae*	*Haemophilus*	0.04 ± 0.04	0.09 ± 0.14	0.10 ± 0.15	0.07 ± 0.10	0.09 ± 0.14	0.13 ± 0.12	0.94	1.30
Proteobacteria	Gammaproteobacteria	Enterobacteriales	*Enterobacteriaceae*	*Citrobacter*	0.17 ± 0.29	0.34 ± 0.62	0.48 ± 0.92	0.40 ± 0.78	0.42 ± 0.82	0.29 ± 0.55	0.99	1.10
Tenericutes	Mollicutes	Anaeroplasmatales	*Anaeroplasmataceae*	*Anaeroplasma*	0.00 ± 0.00	0.00 ± 0.01	0.00 ± 0.01	0.00 ± 0.00	0.00 ± 0.00	0.01 ± 0.01	0.27	2.77
Tenericutes	Mollicutes	RF39	RF39	RF39	0.00 ± 0.00	0.00 ± 0.00	0.00 ± 0.00	0.00 ± 0.01	0.00 ± 0.00	0.00 ± 0.00	0.65	1.64
TM7	TM7-3	TM7-3	TM7-3	TM7-3	0.00 ± 0.01	0.00 ± 0.00	0.00 ± 0.00	0.01 ± 0.01	0.00 ± 0.00	0.01 ± 0.01	0.13	1.90

Abundance with significant differences (p<0.05, q<0.1) is shown in bold letters.
