# Supplementary material for: Effect of storage and DNA extraction method on 16S rRNA-profiled fecal microbiota in Japanese adults
Source: J Clin Biochem Nutr. 2018 Dec 13;64(2):106–11. doi: 10.3164/jcbn.18-84 (PMC6436037; doi:10.3164/jcbn.18-84)
Supplement: Supplemental Table 2 [file jcbn18-84st02.pdf]

Supplemental Table 2. Fecal microbiota composition at genus level in the samples extracted by four different methods

Phylum	Class	Order	Family	Genus	A	B	C	D	*p* values	*q* value
Actinobacteria	Actinobacteria	Actinomycetales	*Actinomycetaceae*	*Actinomyces*	0.04 ± 0.01	0.08 ± 0.07	0.03 ± 0.02	0.03 ± 0.03	0.27	0.94
**Actinobacteria**	**Actinobacteria**	**Bifidobacteriales**	***Bifidobacteriaceae***	***Bifidobacterium***	**12.75 ± 3.64**	**9.78 ± 5.39**	**14.14 ± 2.86**	**1.76 ± 0.76**	**<0.01**	**0.04**
Actinobacteria	Coriobacteriia	Coriobacteriales	*Coriobacteriaceae*	*Collinsella*	2.54 ± 2.36	2.27 ± 2.21	2.88 ± 2.76	0.18 ± 0.10	0.31	0.97
Actinobacteria	Coriobacteriia	Coriobacteriales	*Coriobacteriaceae*	*Eggerthella*	0.11 ± 0.06	0.09 ± 0.03	0.13 ± 0.07	0.02 ± 0.01	0.02	0.37
Actinobacteria	Actinobacteria	Actinomycetales	*Microbacteriaceae*	*Microbacterium*	0.00 ± 0.00	0.01 ± 0.01	0.00 ± 0.00	0.00 ± 0.00	0.42	1.08
Actinobacteria	Actinobacteria	Actinomycetales	*Propionibacteriaceae*	*Propionibacterium*	0.00 ± 0.01	0.00 ± 0.00	0.01 ± 0.02	0.02 ± 0.02	0.14	0.79
Actinobacteria	Actinobacteria	Actinomycetales	*Williamsiaceae*	*Williamsia*	0.00 ± 0.00	0.00 ± 0.00	0.00 ± 0.00	0.00 ± 0.01	0.42	0.98
Bacteroidetes	Bacteroidia	Bacteroidales	[*Paraprevotellaceae*]	[*Prevotella*]	0.01 ± 0.01	0.01 ± 0.01	0.02 ± 0.04	0.03 ± 0.06	0.74	0.96
**Bacteroidetes**	**Bacteroidia**	**Bacteroidales**	***Bacteroidaceae***	***Bacteroides***	**33.47 ± 4.07**	**35.41 ± 4.96**	**23.53 ± 3.17**	**59.01 ± 7.63**	**<0.01**	**<0.01**
Bacteroidetes	Bacteroidia	Bacteroidales	[*Odoribacteraceae*]	*Odoribacter*	0.24 ± 0.29	0.21 ± 0.25	0.17 ± 0.24	0.47 ± 0.58	0.68	0.95
Bacteroidetes	Bacteroidia	Bacteroidales	*Porphyromonadaceae*	*Parabacteroides*	1.86 ± 1.61	1.61 ± 1.49	1.14 ± 1.00	3.09 ± 2.43	0.44	0.94
Bacteroidetes	Bacteroidia	Bacteroidales	*Porphyromonadaceae*	*Porphyromonas*	0.00 ± 0.00	0.00 ± 0.00	0.00 ± 0.00	0.00 ± 0.00	0.58	1.02
Bacteroidetes	Bacteroidia	Bacteroidales	*Prevotellaceae*	*Prevotella*	0.34 ± 0.45	0.33 ± 0.26	0.21 ± 0.26	1.09 ± 0.77	0.08	0.75
Bacteroidetes	Bacteroidia	Bacteroidales	[*Barnesiellaceae*]	Unclassified	0.19 ± 0.38	0.18 ± 0.34	0.09 ± 0.16	0.50 ± 0.97	0.74	0.95
Bacteroidetes	Bacteroidia	Bacteroidales	*Rikenellaceae*	Unclassified	0.42 ± 0.54	0.52 ± 0.66	0.41 ± 0.54	0.84 ± 0.88	0.79	0.96
Bacteroidetes	Bacteroidia	Bacteroidales	S24-7	Unclassified	0.00 ± 0.00	0.04 ± 0.08	0.02 ± 0.04	0.09 ± 0.09	0.29	0.95
Firmicutes	Erysipelotrichi	Erysipelotrichales	*Erysipelotrichaceae*	[*Eubacterium*]	1.48 ± 2.67	0.90 ± 1.54	2.13 ± 3.52	0.13 ± 0.16	0.67	0.97
Firmicutes	Clostridia	Clostridiales	*Lachnospiraceae*	[*Ruminococcus*]	2.66 ± 2.12	2.31 ± 1.84	4.42 ± 3.93	1.90 ± 1.91	0.55	1.05
Firmicutes	Clostridia	Clostridiales	*Veillonellaceae*	*Acidaminococcus*	0.01 ± 0.01	0.01 ± 0.01	0.00 ± 0.00	0.03 ± 0.04	0.10	0.66
Firmicutes	Clostridia	Clostridiales	[*Tissierellaceae*]	*Anaerococcus*	0.00 ± 0.01	0.00 ± 0.01	0.00 ± 0.00	0.00 ± 0.00	0.58	1.00
Firmicutes	Clostridia	Clostridiales	*Eubacteriaceae*	*Anaerofustis*	0.00 ± 0.00	0.00 ± 0.00	0.01 ± 0.01	0.00 ± 0.00	0.09	0.76
Firmicutes	Clostridia	Clostridiales	*Lachnospiraceae*	*Anaerostipes*	0.20 ± 0.26	0.23 ± 0.30	0.23 ± 0.36	0.14 ± 0.17	0.96	1.04
Firmicutes	Clostridia	Clostridiales	*Ruminococcaceae*	*Anaerotruncus*	0.02 ± 0.02	0.02 ± 0.03	0.01 ± 0.01	0.08 ± 0.08	0.15	0.76
Firmicutes	Bacilli	Bacillales	*Bacillaceae*	*Bacillus*	0.02 ± 0.04	0.01 ± 0.01	0.02 ± 0.02	0.00 ± 0.01	0.60	0.99
Firmicutes	Clostridia	Clostridiales	*Lachnospiraceae*	*Blautia*	4.17 ± 2.22	4.01 ± 2.31	6.29 ± 3.72	0.86 ± 0.38	0.05	0.59
Firmicutes	Erysipelotrichi	Erysipelotrichales	*Erysipelotrichaceae*	*Bulleidia*	0.00 ± 0.01	0.00 ± 0.00	0.00 ± 0.00	0.01 ± 0.01	0.54	1.08
Firmicutes	Clostridia	Clostridiales	*Ruminococcaceae*	*Butyricicoccus*	0.64 ± 0.37	0.62 ± 0.26	0.35 ± 0.23	0.28 ± 0.14	0.16	0.78
Firmicutes	Erysipelotrichi	Erysipelotrichales	*Erysipelotrichaceae*	*cc_115*	0.01 ± 0.01	0.02 ± 0.01	0.03 ± 0.05	0.03 ± 0.04	0.76	0.97
Firmicutes	Clostridia	Clostridiales	*Clostridiaceae*	*Clostridium*	0.19 ± 0.20	0.30 ± 0.34	0.25 ± 0.28	0.09 ± 0.09	0.66	0.98
Firmicutes	Erysipelotrichi	Erysipelotrichales	*Erysipelotrichaceae*	*Clostridium*	0.08 ± 0.07	0.12 ± 0.06	0.19 ± 0.15	0.06 ± 0.04	0.20	0.92
Firmicutes	Clostridia	Clostridiales	*Lachnospiraceae*	*Clostridium*	0.25 ± 0.16	0.33 ± 0.13	0.23 ± 0.20	0.43 ± 0.32	0.54	1.06
Firmicutes	Clostridia	Clostridiales	*Ruminococcaceae*	*Clostridium*	0.01 ± 0.01	0.00 ± 0.00	0.01 ± 0.01	0.00 ± 0.00	0.14	0.83
Firmicutes	Erysipelotrichi	Erysipelotrichales	*Erysipelotrichaceae*	*Coprobacillus*	0.32 ± 0.40	0.32 ± 0.43	0.54 ± 0.56	0.05 ± 0.06	0.43	0.95
Firmicutes	Clostridia	Clostridiales	*Lachnospiraceae*	*Coprococcus*	2.42 ± 1.25	2.25 ± 1.21	1.30 ± 1.24	0.68 ± 0.36	0.12	0.72
Firmicutes	Clostridia	Clostridiales	*Dehalobacteriaceae*	*Dehalobacterium*	0.00 ± 0.00	0.00 ± 0.00	0.00 ± 0.00	0.00 ± 0.00	1.00	1.03
Firmicutes	Clostridia	Clostridiales	*Veillonellaceae*	*Dialister*	0.48 ± 0.66	0.52 ± 0.86	0.50 ± 0.70	0.35 ± 0.24	0.98	1.04
Firmicutes	Clostridia	Clostridiales	*Lachnospiraceae*	*Dorea*	0.76 ± 0.65	0.70 ± 0.70	1.11 ± 0.98	0.37 ± 0.49	0.57	1.03
Firmicutes	Bacilli	Lactobacillales	*Enterococcaceae*	*Enterococcus*	0.02 ± 0.03	0.00 ± 0.00	0.04 ± 0.05	0.02 ± 0.02	0.55	1.04
Firmicutes	Clostridia	Clostridiales	*Ruminococcaceae*	*Faecalibacterium*	9.03 ± 3.40	10.21 ± 4.61	8.13 ± 3.62	6.68 ± 4.02	0.64	0.98
Firmicutes	Bacilli	Lactobacillales	*Carnobacteriaceae*	*Granulicatella*	0.00 ± 0.01	0.01 ± 0.01	0.01 ± 0.01	0.01 ± 0.01	0.77	0.97
Firmicutes	Erysipelotrichi	Erysipelotrichales	*Erysipelotrichaceae*	*Holdemania*	0.04 ± 0.05	0.05 ± 0.06	0.03 ± 0.04	0.03 ± 0.06	0.95	1.03
Firmicutes	Clostridia	Clostridiales	*Lachnospiraceae*	*Lachnospira*	1.04 ± 1.23	0.90 ± 1.06	0.95 ± 1.35	0.68 ± 0.49	0.97	1.03
Firmicutes	Bacilli	Lactobacillales	*Lactobacillaceae*	*Lactobacillus*	0.04 ± 0.04	0.04 ± 0.03	0.10 ± 0.17	0.13 ± 0.18	0.63	0.98
Firmicutes	Bacilli	Lactobacillales	*Streptococcaceae*	*Lactococcus*	0.01 ± 0.02	0.01 ± 0.01	0.03 ± 0.07	0.00 ± 0.00	0.55	1.02
Firmicutes	Bacilli	Lactobacillales	*Leuconostocaceae*	*Leuconostoc*	0.01 ± 0.01	0.00 ± 0.00	0.01 ± 0.02	0.00 ± 0.00	0.59	0.98
Firmicutes	Clostridia	Clostridiales	*Veillonellaceae*	*Megamonas*	0.00 ± 0.01	0.01 ± 0.01	0.01 ± 0.02	0.08 ± 0.08	0.05	0.65
Firmicutes	Clostridia	Clostridiales	*Veillonellaceae*	*Megasphaera*	0.50 ± 0.89	0.51 ± 0.92	0.34 ± 0.63	0.05 ± 0.05	0.78	0.97
Firmicutes	Clostridia	Clostridiales	*Ruminococcaceae*	*Oscillospira*	1.64 ± 1.19	2.28 ± 1.63	1.26 ± 0.93	1.63 ± 1.25	0.72	0.97
Firmicutes	Clostridia	Clostridiales	[*Tissierellaceae*]	*Parvimonas*	0.01 ± 0.01	0.00 ± 0.00	0.00 ± 0.00	0.00 ± 0.01	0.72	0.99
Firmicutes	Clostridia	Clostridiales	[*Tissierellaceae*]	*Peptoniphilus*	0.00 ± 0.00	0.00 ± 0.01	0.00 ± 0.00	0.01 ± 0.01	0.68	0.96
Firmicutes	Clostridia	Clostridiales	*Veillonellaceae*	*Phascolarctobacterium*	0.46 ± 0.92	0.51 ± 0.97	0.25 ± 0.47	0.26 ± 0.43	0.94	1.03
Firmicutes	Clostridia	Clostridiales	*Eubacteriaceae*	*Pseudoramibacter_Eubacterium*	0.02 ± 0.04	0.02 ± 0.04	0.00 ± 0.00	0.00 ± 0.01	0.65	0.98
Firmicutes	Clostridia	Clostridiales	*Lachnospiraceae*	*Roseburia*	1.20 ± 1.10	1.61 ± 1.25	1.40 ± 1.05	1.07 ± 0.75	0.89	1.01
Firmicutes	Clostridia	Clostridiales	*Ruminococcaceae*	*Ruminococcus*	2.50 ± 3.86	2.68 ± 4.19	2.16 ± 3.81	3.10 ± 4.84	0.99	1.03
Firmicutes	Clostridia	Clostridiales	*Clostridiaceae*	SMB53	0.47 ± 0.31	0.36 ± 0.20	0.18 ± 0.10	0.24 ± 0.09	0.21	0.92
Firmicutes	Bacilli	Lactobacillales	*Streptococcaceae*	*Streptococcus*	0.63 ± 0.42	0.51 ± 0.53	0.77 ± 0.57	0.49 ± 0.32	0.83	0.97
Firmicutes	Bacilli	Turicibacterales	*Turicibacteraceae*	*Turicibacter*	0.13 ± 0.11	0.07 ± 0.08	0.09 ± 0.11	0.05 ± 0.06	0.67	0.98
Firmicutes	Clostridia	Clostridiales	[*Mogibacteriaceae*]	Unclassified	0.04 ± 0.05	0.03 ± 0.04	0.05 ± 0.04	0.01 ± 0.01	0.60	0.97
Firmicutes	Clostridia	Clostridiales	*Christensenellaceae*	Unclassified	0.00 ± 0.01	0.01 ± 0.01	0.01 ± 0.01	0.00 ± 0.01	0.81	0.98
Firmicutes	Clostridia	Clostridiales	*Clostridiaceae*	Unclassified	0.09 ± 0.12	0.14 ± 0.23	0.19 ± 0.28	0.26 ± 0.42	0.85	0.98
Firmicutes	Erysipelotrichi	Erysipelotrichales	*Erysipelotrichaceae*	Unclassified	0.53 ± 0.49	0.39 ± 0.37	1.02 ± 0.93	0.16 ± 0.14	0.21	0.90
Firmicutes	Bacilli	Gemellales	*Gemellaceae*	Unclassified	0.01 ± 0.02	0.01 ± 0.01	0.01 ± 0.01	0.01 ± 0.02	0.83	0.98
Firmicutes	Clostridia	Clostridiales	*Lachnospiraceae*	Unclassified	4.18 ± 1.68	4.78 ± 2.04	7.39 ± 2.69	1.65 ± 0.72	0.01	0.20
Firmicutes	Clostridia	Clostridiales	*Ruminococcaceae*	Unclassified	2.51 ± 2.29	2.95 ± 2.91	1.55 ± 1.28	0.55 ± 0.32	0.35	0.97
Firmicutes	Clostridia	Clostridiales	Unclassified	Unclassified	0.48 ± 0.45	0.48 ± 0.47	0.27 ± 0.21	0.59 ± 0.71	0.83	0.99
Firmicutes	Clostridia	Clostridiales	*Veillonellaceae*	*Veillonella*	3.91 ± 3.40	3.25 ± 2.63	2.80 ± 2.82	2.62 ± 1.75	0.91	1.01
Firmicutes	Clostridia	Clostridiales	[*Tissierellaceae*]	WAL_1855D	0.01 ± 0.01	0.01 ± 0.01	0.00 ± 0.01	0.00 ± 0.00	0.33	0.97
Fusobacteria	Fusobacteriia	Fusobacteriales	*Fusobacteriaceae*	*Fusobacterium*	0.03 ± 0.03	0.02 ± 0.03	0.02 ± 0.02	0.07 ± 0.04	0.10	0.75
Fusobacteria	Fusobacteriia	Fusobacteriales	*Leptotrichiaceae*	*Leptotrichia*	0.00 ± 0.01	0.00 ± 0.01	0.00 ± 0.00	0.02 ± 0.02	0.28	0.95
Proteobacteria	Deltaproteobacteria	Desulfovibrionales	*Desulfovibrionaceae*	*Bilophila*	0.19 ± 0.22	0.11 ± 0.13	0.06 ± 0.07	0.12 ± 0.17	0.73	0.97
Proteobacteria	Alphaproteobacteria	Rhizobiales	*Bradyrhizobiaceae*	*Bradyrhizobium*	0.00 ± 0.00	0.03 ± 0.06	0.00 ± 0.00	0.01 ± 0.01	0.43	0.97
Proteobacteria	Epsilonproteobacteria	Campylobacterales	*Campylobacteraceae*	*Campylobacter*	0.00 ± 0.00	0.00 ± 0.01	0.00 ± 0.00	0.01 ± 0.01	0.25	1.02
Proteobacteria	Gammaproteobacteria	Enterobacteriales	*Enterobacteriaceae*	*Citrobacter*	0.02 ± 0.04	0.03 ± 0.04	0.02 ± 0.02	0.08 ± 0.08	0.27	0.97
Proteobacteria	Betaproteobacteria	Burkholderiales	*Comamonadaceae*	*Delftia*	0.00 ± 0.00	0.01 ± 0.01	0.00 ± 0.00	0.02 ± 0.03	0.40	1.05
Proteobacteria	Gammaproteobacteria	Enterobacteriales	*Enterobacteriaceae*	*Escherichia*	1.29 ± 1.19	1.48 ± 1.47	0.56 ± 0.51	1.55 ± 1.13	0.59	1.00
Proteobacteria	Gammaproteobacteria	Pasteurellales	*Pasteurellaceae*	*Haemophilus*	0.14 ± 0.12	0.21 ± 0.21	0.06 ± 0.03	0.42 ± 0.27	0.07	0.74
Proteobacteria	Gammaproteobacteria	Enterobacteriales	*Enterobacteriaceae*	*Klebsiella*	0.05 ± 0.07	0.09 ± 0.11	0.02 ± 0.02	0.11 ± 0.06	0.34	0.99
Proteobacteria	Gammaproteobacteria	Xanthomonadales	*Xanthomonadaceae*	*Lysobacter*	0.00 ± 0.00	0.00 ± 0.00	8.48 ± 7.96	0.00 ± 0.00	0.02	0.34
Proteobacteria	Alphaproteobacteria	Rhizobiales	*Methylobacteriaceae*	*Methylobacterium*	0.00 ± 0.00	0.13 ± 0.22	0.00 ± 0.00	0.00 ± 0.00	0.31	0.94
Proteobacteria	Betaproteobacteria	Neisseriales	*Neisseriaceae*	*Neisseria*	0.00 ± 0.00	0.00 ± 0.01	0.00 ± 0.01	0.04 ± 0.03	0.01	0.17
Proteobacteria	Betaproteobacteria	Burkholderiales	*Comamonadaceae*	*Pelomonas*	0.00 ± 0.00	0.08 ± 0.16	0.00 ± 0.00	0.00 ± 0.00	0.42	1.00
Proteobacteria	Gammaproteobacteria	Pseudomonadales	*Pseudomonadaceae*	*Pseudomonas*	0.33 ± 0.62	0.36 ± 0.61	0.04 ± 0.02	0.46 ± 0.34	0.63	0.99
Proteobacteria	Gammaproteobacteria	Enterobacteriales	*Enterobacteriaceae*	*Serratia*	0.00 ± 0.01	0.01 ± 0.01	0.00 ± 0.01	0.00 ± 0.00	0.71	0.99
Proteobacteria	Alphaproteobacteria	Sphingomonadales	*Sphingomonadaceae*	*Sphingomonas*	0.00 ± 0.00	0.00 ± 0.00	0.00 ± 0.00	0.01 ± 0.01	0.42	1.02
Proteobacteria	Gammaproteobacteria	Xanthomonadales	*Xanthomonadaceae*	*Stenotrophomonas*	0.03 ± 0.07	0.05 ± 0.07	0.01 ± 0.01	0.01 ± 0.02	0.58	1.03
Proteobacteria	Betaproteobacteria	Burkholderiales	*Alcaligenaceae*	*Sutterella*	2.56 ± 1.43	2.26 ± 1.37	1.14 ± 0.62	3.85 ± 1.90	0.10	0.79
Proteobacteria	Alphaproteobacteria	Rickettsiales	*mitochondria*	Unclassified	0.02 ± 0.02	0.01 ± 0.01	0.00 ± 0.01	0.00 ± 0.01	0.26	1.00
Proteobacteria	Alphaproteobacteria	Rhizobiales	Unclassified	Unclassified	0.00 ± 0.00	0.00 ± 0.01	0.00 ± 0.00	0.00 ± 0.00	0.42	1.05
Proteobacteria	Unclassified	Unclassified	Unclassified	Unclassified	0.00 ± 0.00	0.01 ± 0.01	0.00 ± 0.00	0.00 ± 0.00	0.52	1.05
Proteobacteria	Alphaproteobacteria	RF32	Unclassified	Unclassified	0.06 ± 0.12	0.03 ± 0.07	0.03 ± 0.07	0.09 ± 0.17	0.89	1.01
Proteobacteria	Alphaproteobacteria	Rhizobiales	*Xanthobacteraceae*	*Xanthobacter*	0.00 ± 0.00	0.00 ± 0.00	0.00 ± 0.00	0.00 ± 0.00	1.00	1.02
Spirochaetes	[Brachyspirae]	[Brachyspirales]	*Brachyspiraceae*	*Brachyspira*	0.02 ± 0.02	0.00 ± 0.01	0.02 ± 0.02	0.08 ± 0.07	0.10	0.70
TM7	TM7-3	Unclassified	Unclassified	Unclassified	0.00 ± 0.01	0.00 ± 0.01	0.01 ± 0.01	0.01 ± 0.01	0.51	1.06
Verrucomicrobia	Verrucomicrobiae	Verrucomicrobiales	*Verrucomicrobiaceae*	*Akkermansia*	0.01 ± 0.01	0.00 ± 0.00	0.01 ± 0.01	0.02 ± 0.03	0.26	1.01

Abundance with significant differences (*p*<0.05, *q*<0.1) is shown in bold letters.
